# Loxl3 Affects Palatal Shelf Elevation by Regulating Cell Proliferation and Collagen Deposition

**DOI:** 10.3390/ijms26104815

**Published:** 2025-05-17

**Authors:** Ziyi Liu, Fan Mo, Xinyu Dong, Ge Chen, Jiangang Gao, Jian Zhang

**Affiliations:** School of Life Science, Shandong University, Qingdao 266237, China; liuziyi@sdfmu.edu.cn (Z.L.); skylight234@163.com (F.M.); dxinyu77@163.com (X.D.); chenge2577@163.com (G.C.)

**Keywords:** Lysyl oxidase-like 3, palatal shelves, cell proliferation, collagen deposition

## Abstract

Cleft palate is one of the most common congenital abnormalities and one of the main symptoms of Stickler syndrome. Secondary palate development is a complex multi-step process that involves raising the palatal frame from a vertical to a horizontal position. Lysyl oxidase-like 3 (*LOXL3*), a member of the lysyl oxidase family responsible for the crosslinking in collagen, is also one of the mutated genes detected in Stickler syndrome. Loss of *Loxl3* causes delayed palatal shelf elevation, which in turn resulted in cleft palate. However, the precise mechanisms of palatal shelf delayed elevation remain unclear. In this study, we deeply investigated the mechanism of *Loxl3* induced delayed elevation in palatal shelves. We found that *Loxl3* deficiency caused reduced cell proliferation in both medial and posterior palatal mesenchyme through BrdU labeling and Western blot analysis (*p* < 0.05, *p* < 0.01), decreased migration of palatal mesenchymal cells through cell scratch assay (*p* < 0.05), and decreased expression of genes associated with proliferation through Western blot analysis (*p* < 0.05, *p* < 0.01) at E14. We found that the specific deletion of Loxl3 in the palatal mesenchyme resulted in delayed elevation but normal fusion of palatal shelves, also reduced cell proliferation and collagen fibers deposition in medial palatal mesenchyme through BrdU labeling and histological analysis (*p* < 0.05, *p* < 0.01). Thus, our data suggest that Loxl3 regulates cell proliferation and collagen fibers deposition in the palatal mesenchyme, thus controlling palatal shelf elevation.

## 1. Introduction

Cleft lip and palate or isolated cleft palate are one of the most common human congenital craniofacial birth defects seen with a prevalence ranging from 1500 to 2500 newborns in different nations and regions under different racial backgrounds [1,2]. Mammalian palatogenesis is an accurate and complex embryonic developmental process leading to the complicated pathology of cleft palate. It is generally accepted that the palatal development involves palatal shelf initiation, vertical growth, reorientation, horizontal growth and fusion, and failure in any of these stages mediated through genetic and environmental factors could lead to cleft palate [3,4]. To research the etiological basis of cleft palate, more than 250 genes associated with cleft palate have been identified through extensive human genetic studies and analyses of laboratory mice with induced mutations or targeted tissue-specific gene inactivation [5]. Stickler syndrome is a connective tissue disease involving bone, eye, and facial structures [6]. Studies have shown that cleft palate is a prominent phenotype of Stickler syndrome, and *LOXL3* mutation can cause Stickler syndrome [7,8]. LOXL3 in a member of Lysyl oxidase (LOX) family, which are copper-dependent amine oxidases, catalyzing the oxidative deamination of lysine and hydroxylysine residues, generating covalent cross-links which stabilize polymeric elastin and collagen fibers in the extracellular matrix (ECM) [9]. In our previous study, we found that *Loxl3* deletion caused delayed palatal shelf elevation and eventually form cleft palate [10]. Secondary palate development begins with the formation of paired palatal frames on E11.5, followed by vertical growth on both sides of the tongue, from E12.5 to E13.5, and rising to horizontal between E14 and E14.5 [11]. Cell proliferation and migration play an important role in palate development [12,13,14]. Various collagen proteins are highly expressed in the palate and are reshaped dynamically during the formation of the palate [15,16]. Our previous study suggested that *Loxl3* deletion affected collagen resemble, resulting in delayed palatal shelf elevation [10]. However, we believe that the occurrence of delayed palatal shelf elevation may also be related to other factors. To explore this problem, we conducted in vitro and in vivo experiments to explore the role of *Loxl3* in palatal shelf elevation. We further identify *Loxl3* deficiency reduced *Osr2*, *Fgf10*, *Smo* and *Shh* signal transduction and reduced levels of cell proliferation and migration in the palatal mesenchyme in E14. Moreover, the loss of *Loxl3* affects cell proliferation and collagen fibers deposition in *Col2a1-Cre; Loxl3^f/f^* mice, which affects palatal shelf elevation. Exploring the relationship between Loxl3 and cleft palate may be helpful for the clinical research of targeted drugs for the treatment of cleft palate.

## 2. Result

### 2.1. Delayed Palatal Shelf Elevation Due to Loxl3 Deletion Occurred Between E14 and E14.5

**Figure 1:**
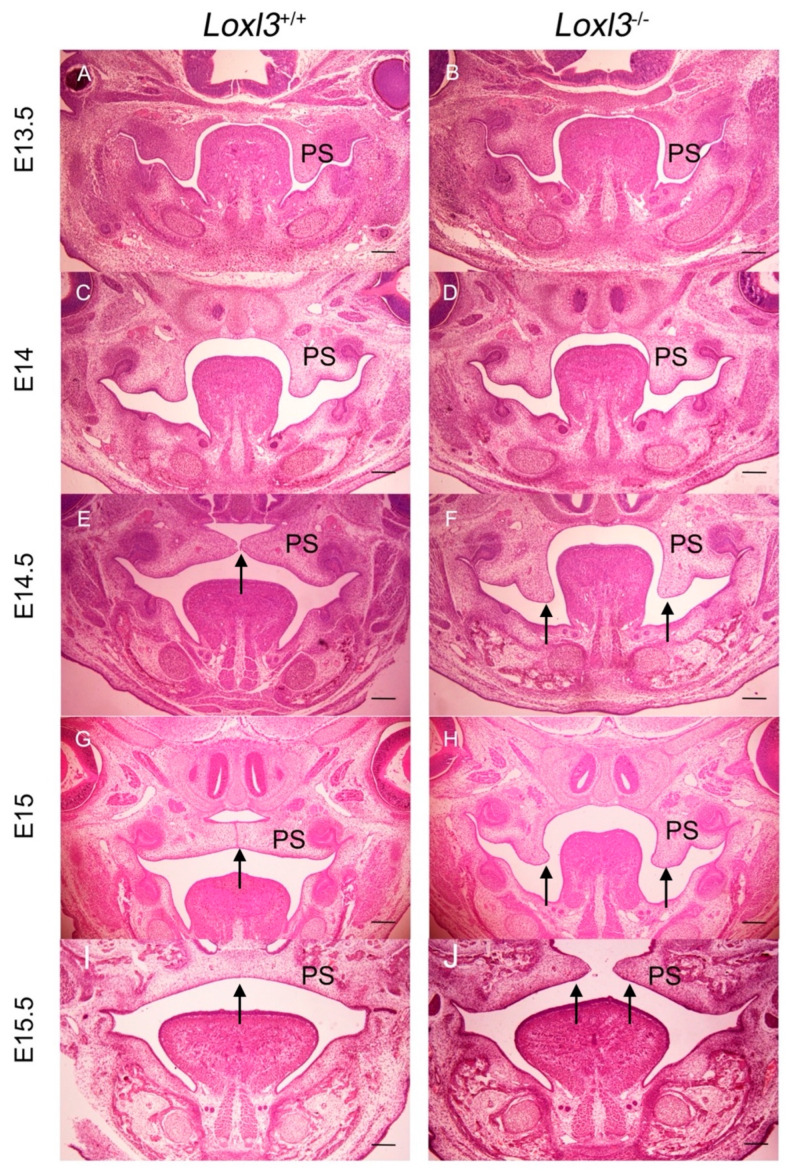
Ablation of *Loxl3* led to delayed elevation of palatal shelves between E14 and E14.5. (**A**,**B**) Palatal shelves of *Loxl3*^+/+^ and *Loxl3*^−/−^ embryos at E13.5. (**C**,**D**) Palatal shelves of *Loxl3*^+/+^ and *Loxl3*^−/−^ embryos at E14. (**E**,**F**) Palatal shelves of *Loxl3*^+/+^ and *Loxl3*^−/−^ embryos at E14.5. (**G**,**H**) Palatal shelves of *Loxl3*^+/+^ and *Loxl3*^−/−^ embryos at E15. (**I**,**J**) Palatal shelves of *Loxl3*^+/+^ and *Loxl3*^−/−^ embryos at E15.5. PS: palatal shelves. Bar: 500 μm.

As our previous study showed, all of the *Loxl3*^−/−^ mice exhibited retarded elevation of palatal shelves between E13.5 and E14.5 [10]. In this experiment, we found that the delayed palatal shelf elevation occurred between E14 and E14.5 in *Loxl3*^−/−^ mice (Figure 1A–F, black arrow). The wild-type mice had completed the adhesion and fusion of palatal shelves, but the palatal shelves of *Loxl3*^−/−^ mice just began to lift at E15 (Figure 1G,H, black arrow). Then, the palatal shelves of *Loxl3*^−/−^ mice was basically raised, but fusion did not occur at E15.5 (Figure 1I,J, black arrow), keeping the state of palate to the birth of mice. 92.31% (48/52) of *Loxl3*^−/−^ mice showed cleft palate.

### 2.2. Loxl3 Deletion Caused Reduced Cell Proliferation and Expression of Proliferation-Related Genes In Vivo

**Figure 2:**
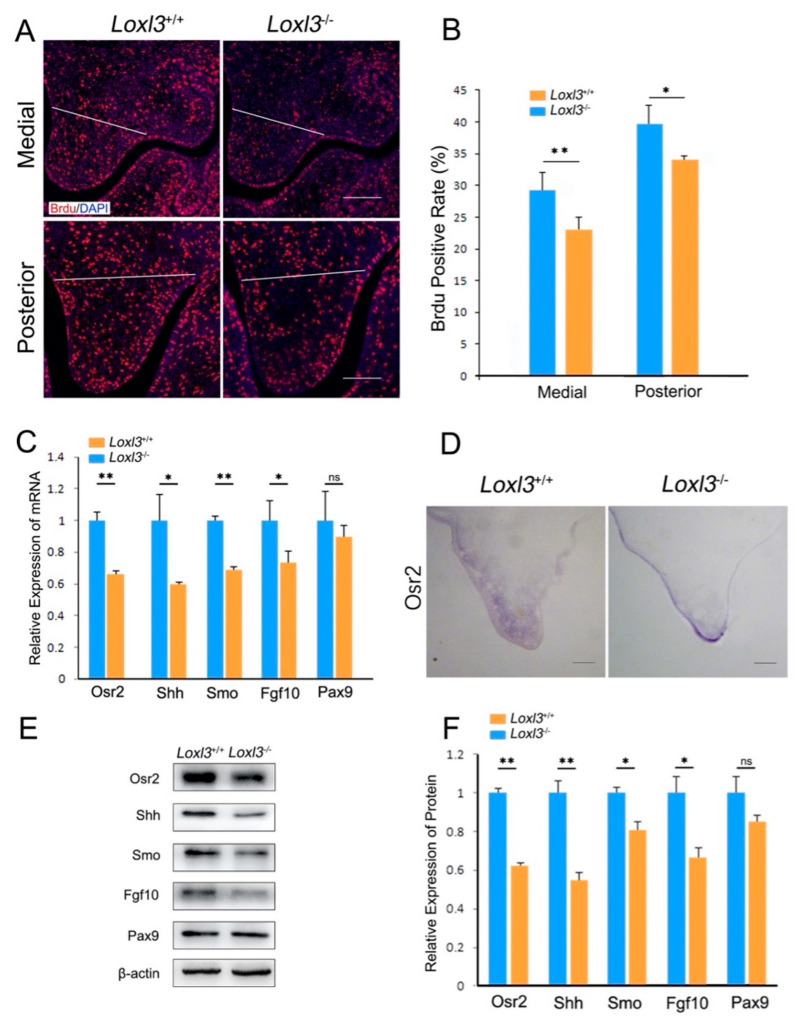
*Loxl3*^−/−^ embryos exhibited reduced cell proliferation and reduced expression of several proliferation-related genes at E14. (**A**) BrdU labeling assay for medial and posterior palatal shelves of *Loxl3*^−/−^ and *Loxl3*^+/+^ embryos at E14. White lines indicate the palatal process region. Bar: 200 μm. (**B**) Statistical analyses of cell proliferation in palatal shelves. (**C**) Comparison of Osr2, Shh, Smo, Fgf10 and *Pax9* mRNA expression in palatal shelves of *Loxl3*^−/−^ and *Loxl3*^+/+^ embryos at E14. (**D**) In situ hybridization detection of *Osr2* mRNA (positive signal) in palatal shelves of *Loxl3*^−/−^ and *Loxl3*^+/+^ embryos at E14. Bar: 200 μm. (**E**,**F**) Western blot analysis of Osr2, Shh, Smo, Fgf10 and Pax9 expression in the palatal shelves of *Loxl3*^−/−^ and *Loxl3*^+/+^ embryos at E14. *n* = 5, * *p* < 0.05, ** *p* < 0.01, ns = not significant. mean ± SEM.

Previous studies had shown that palatal shelf outgrowth is associated with reduced cell proliferation [12,13]. To investigate whether the delayed elevation of palatal shelves in *Loxl3*^−/−^ mice embryos was due to the abnormal cell proliferation, we detected the cell proliferation of the palatal mesenchymal cells in E14 mice by Brdu incorporation. At E14, compared with cell proliferation in the medial and posterior palate of wild-type mice, the *Loxl3*^−/−^ mice exhibited 6.26% (29.15–22.89%) reduction (*p* < 0.01) in cell proliferation index in the medial palatal mesenchyme and 5.61% (39.45–33.84%) reduction (*p* < 0.05) in the posterior palatal mesenchyme (Figure 2A,B). Thus, besides the impaired deposition of mature collagen fibers [10], the reduction in cell proliferation of palatal mesenchyme probably aggravated the retarded elevation at E14.

To investigate the molecular mechanism that mediates the reduced cell proliferation in palatal mesenchyme of *Loxl3*^−/−^ mice, some molecular factors associated with proliferation, such as Osr2, Fgf10, Pax9, Shh and Smo, were analyzed in the developing palatal shelves of *Loxl3*^−/−^ and *Loxl3*^+/+^ embryos at E14 according to previous studies [17,18,19]. These proliferation-related genes form complex molecular networks in palate development and control palate development [17]. The *Osr2* mRNA expression was significantly reduced in the developing palate in the *Loxl3*^−/−^ embryos, as detected by both real-time RT-PCR and section in situ hybridization assays (*p* < 0.01, Figure 2C,D). Moreover, the expressions of *Shh*, *Smo*, *Fgf10* mRNAs was also obviously downregulated in the developing palatal shelves of *Loxl3*^−/−^ mice compared with wild-type littermates (*p* < 0.05, *p* < 0.01, Figure 2C). The expression of the Osr2, Shh, Smo and Fgf10 proteins was significantly decreased in the developing palatal shelves of *Loxl3*^−/−^ mice at E14 (*p* < 0.05, *p* < 0.01, Figure 2E,F). In palate development, Pax9 regulation is involved in regulation of the Fgf10, Shh, Osr2 pathways [17]. The expression of Pax9 in the developing palatal shelves of *Loxl3*^−/−^ mice was also downregulated, but the difference is not significant.

### 2.3. Loxl3 Deletion Resulted in Reduced Cell Proliferation and Migration In Vitro

**Figure 3:**
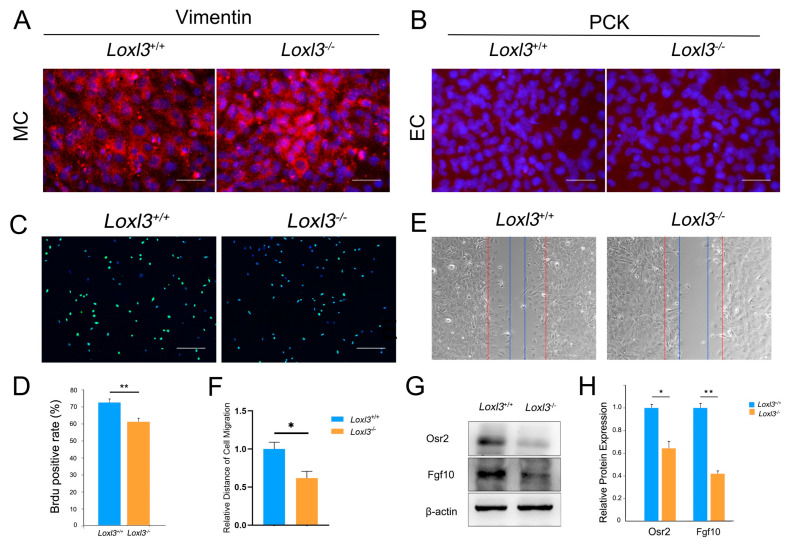
Ablation of *Loxl3* resulted in decreased cell proliferation, migration, and Osr2 and Fgf10 expression in primary palatal mesenchymal cells of E14. (**A**,**B**) Immunofluorescence staining of Vimentin and pan-cytokeratins (PCK). The primary cultured mesenchymal cell (MC) showed positive staining of Vimentin and null staining of PCK. Bar: 200 μm. (**C**,**D**) BrdU labeling assay for the primary cultured MC. Bar: 200 μm. (**E**,**F**) Scratch assay of the cultured MC. Scratches of 1000 µm wide were made in the cultured MC layer from E14 *Loxl3*^−/−^ and *Loxl3*^+/+^ palatal shelves (red line). After 6 h of cell culture, the width of the scratches in the *Loxl3*^+/+^ MC was smaller than that in *Loxl3*^−/−^ MC (blue line). Bar: 100 μm. (**G**,**H**) Western blot analysis of Osr2 and Fgf10 expression in the primary cultured MC of *Loxl3*^−/−^ and *Loxl3*^+/+^ palatal shelves. *n* = 5, * *p* < 0.05, ** *p* < 0.01, mean ± SEM.

Osr2 and Fgf10 in palatogenesis have been deeply studied, and shown to play an important role in the development of palate [20,21]. We observed decreased expression of Osr2 and Fgf10 and decreased cell proliferation in *Loxl3*^−/−^ embryonic palatal shelves in vivo at E14, in order to verify whether Loxl3 affects mesenchymal cell proliferation by regulating Osr2 and Fgf10 in the palatal mesenchyme—we also designed an in vitro experiment. We separated mesenchyme of palatal shelves in embryos at E14 by Dispase II and primary cultured the mesenchymal cell (MC), confirming which by the Vimentin (mesenchymal cell marker) and epithelial cell marker pan-cytokeratins (PCK, epithelial cell marker) through immunofluorescence (Figure 3A,B). Then, we detected the cell proliferation of the primary cultured MC by Brdu incorporation and the MC of *Loxl3*^−/−^ mice embryos exhibited 10% reduction (*p* < 0.01) in cell proliferation index (*p* < 0.01, Figure 3C,D). Interestingly, scratch assay was performed to examine the mobility of *Loxl3*^−/−^ MC. After 6 h in vitro culture, the MC relative migration distance in *Loxl3*^−/−^ MC was significantly shorter than that in *Loxl3*^+/+^ MC (*p* < 0.05, Figure 3E,F). Moreover, the real-time RT-PCR (Supplementary Figure S1) and Western blot analysis were also performed to examine the Osr2 and Fgf10 expression, which were reduced in primary cultured MC of *Loxl3*^−/−^ mice embryos, in accordance with the results in vivo (*p* < 0.05, *p* < 0.01, Figure 3G,H).

### 2.4. Col2a1-Cre Mediated Ablation of the Loxl3 in the Palatal Mesenchyme

**Figure 4:**
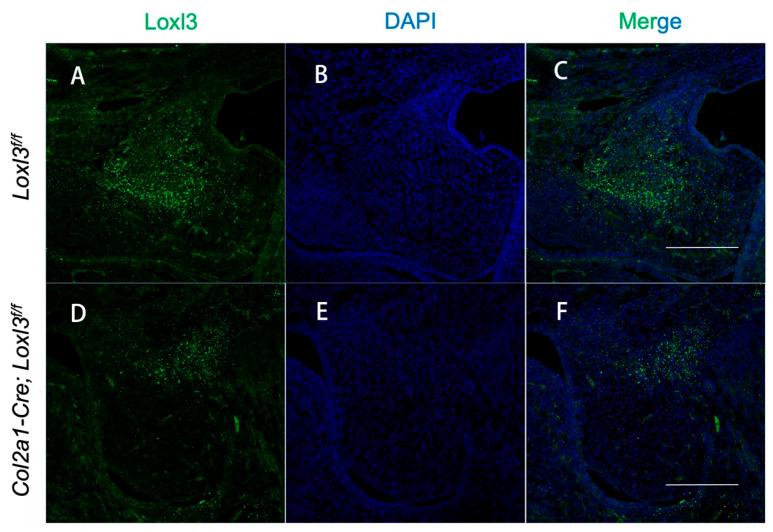
Ablation of *Loxl3* in the palatal mesenchyme was mediated by Col2a1-Cre at E14.5. (**A**–**F**) The expression of Loxl3 (green) was visibly reduced in mesenchyme of *Col2a1-Cre; Loxl3^f/f^* palatal shelves at E14.5. Nuclei, blue. Bar: 200 μm.

To further investigate the role of Loxl3 in palatal mesenchyme, Loxl3 conditional knockout mice were generated with the Cre-loxP system. Homozygous mice (*Loxl3^f/f^*) carrying the floxed *Loxl3* allele were crossed with Col2a1-Cre mice [22]. We analyzed the expression pattern of Col2a1-Cre recombinase with the *Rosa26-tdTomato* reporter mouse strain [23]. Embryonic palate tissues from Col2a1-Tomato mice were analyzed for Cre activity by immunofluorescence staining. As the results shown, Cre activity was strongly positive in mesenchyme of palatal shelves at E14.5 (Supplementary Figure S2). Compared with *Loxl3^f/f^* embryos, the expression of Loxl3 in palatal mesenchyme of *Col2a1-Cre; Loxl3^f/f^* embryos were significantly decreased in palatal mesenchyme at E14.5 (Figure 4A–F).

### 2.5. Col2a1-Cre-Mediated Loxl3 Ablation Led to Delayed Elevation but Normal Fusion of Palatal Shelves

**Figure 5:**
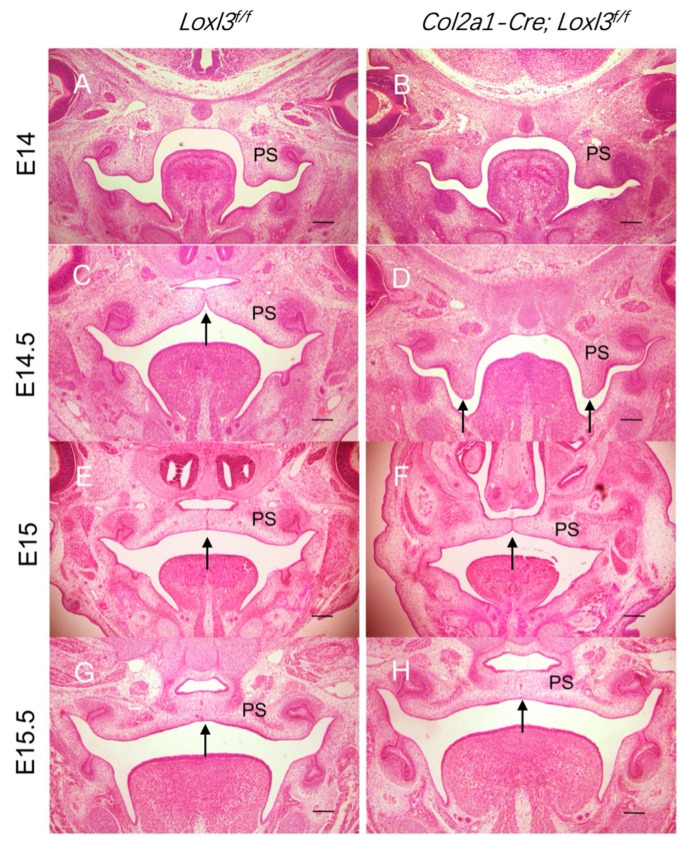
Palatal shelves of *Col2a1-Cre; Loxl3^f/f^* embryos exhibited delayed elevation but normal fusion. (**A**,**B**) Palatal shelves of *Loxl3^f/f^* and *Col2a1-Cre; Loxl3^f/f^* embryos at E14. (**C**,**D**) Palatal shelves of *Loxl3^f/f^* and *Col2a1-Cre; Loxl3^f/f^* embryos at E14.5. (**E**,**F**) Palatal shelves of *Loxl3^f/f^* and *Col2a1-Cre; Loxl3^f/f^* embryos at E15. (**G**,**H**) Palatal shelves of *Loxl3^f/f^* and *Col2a1-Cre; Loxl3^f/f^* embryos at E15.5. PS: palatal shelves. Bar: 500 μm.

There was no significant difference between *Col2a1-Cre; Loxl3^f/f^* mouse palatal shelves and wild-type mouse palatal shelves at E14 (Figure 5A,B). Unlike wild-type mice, palatal shelves of approximately 90% (44/49) of *Col2a1-Cre; Loxl3^f/f^* mice failed to elevate at E14.5 (Figure 5C,D, black arrow), which were consistent with *Loxl3*^−/−^ mice. However, about 90% (40/44) of the palatal shelves of *Col2a1-Cre; Loxl3^f/f^* mice were elevated to horizontal position and grew to meet at midline at E15 (Figure 5E,F, black arrow), then the fusion of epithelial layers was completed normally at E15.5 (Figure 5G,H, black arrow), which were similar to wild-type mice. As a result, none of *Col2a1-Cre; Loxl3^f/f^* embryos had cleft palate. This suggested that delayed palatal shelf elevation does not necessarily lead to cleft palate, and the palatal shelf is rapidly elevated in a short period of time to keep up with normal palatal development.

### 2.6. Col2a1-Cre; Loxl3^f/f^ Embryos Exhibited Reduced Cell Proliferation and Collagen Deposition

**Figure 6:**
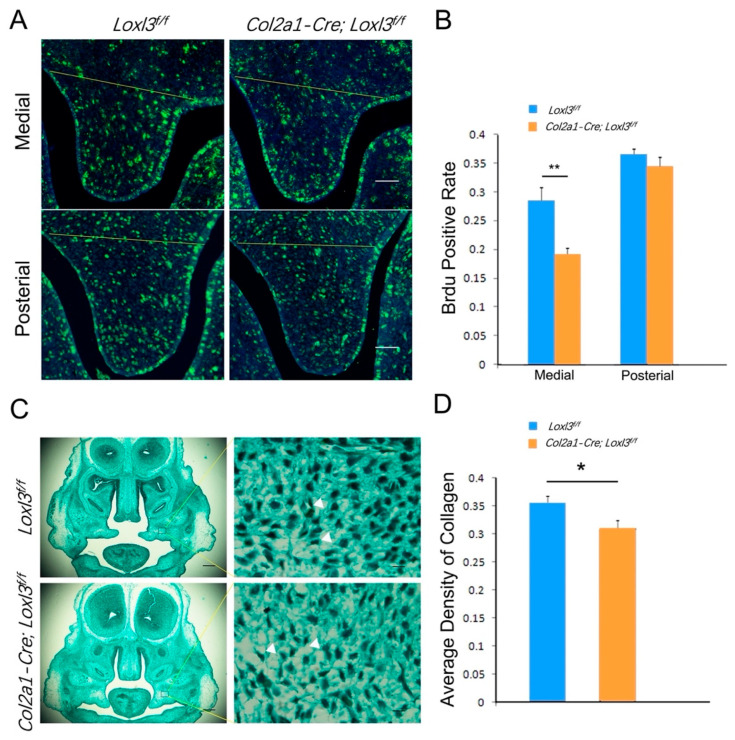
*Col2a1-Cre; Loxl3^f/f^* palatal mesenchyme exhibited reduced cell proliferation at E14 and reduced collagen fibers density at E14.5. (**A**) BrdU labeling assay for medial and posterior palatal shelves of *Loxl3^f/f^* and *Col2a1-Cre; Loxl3^f/f^* embryos at E14. Yellow lines indicate the palatal process region. Bar: 200 μm. (**B**) Statistical analyses of cell proliferation in palatal shelves. (**C**,**D**) Representative ×4 and ×40 images of trichrome-stained embryonic palatal mesenchyme sections of *Loxl3^f/f^* mice at E14.5. Left bar: 200 μm. Right bar: 10 μm. Collagen fibers (white triangles) were stained. *n* = 5, * *p* < 0.05, ** *p* < 0.01, mean ± SEM.

We also detected the cell proliferation of the palatal mesenchymal cells of *Loxl3^f/f^* and *Col2a1-Cre; Loxl3^f/f^* embryos at E14 by Brdu incorporation and the *Col2a1-Cre; Loxl3^f/f^* embryos exhibited 7% reduction (*p* < 0.01) in cell proliferation index in the medial palatal mesenchyme (Figure 6A). However, the cell proliferation index in the posterior palatal shelf cells did not change significantly compared with *Loxl3^f/f^* embryos (Figure 6A). The statistical results showed that the cell proliferation rate decreased significantly only in the middle palatal mesenchyme (*p* < 0.01, Figure 6B). This reduction in cell proliferation of medial palatal shelves probably results in the appearance of retarded elevation at E14, consistent with *Loxl3*^−/−^ embryos.

In our previous study, we had examined the collagen fibers in palatal shelves of mice at E14.5, and Masson’s trichrome staining showed a significant decrease in collagen fibers deposition in *Loxl3*^−/−^ embryonic palatal shelves [10]. In this study, sections of E14.5 embryonic palatal shelves from *Loxl3^f/f^* and *Col2a1-Cre; Loxl3^f/f^* embryos were also stained with Masson’s trichrome staining to visualize mature collagen fibers deposition. We found that there was also a significant decrease in collagen fiber density in *Col2a1-Cre; Loxl3^f/f^* palatal shelves compared with that in *Loxl3^f/f^* palatal mesenchyme at E14.5 (*p* < 0.05, Figure 6C,D).

## 3. Discussion

Stickler syndrome is a collagenopathy caused by a mutation in the *LOXL3* gene with a distinct cleft palate [6,24]. Cleft palate is a common congenital deformity that can be caused by disruption at any stage of the formation of the palate, including palatal shelf growth, elevation, adhesion, and fusion. In our previous study, we proposed that the loss of *Loxl3* resulted in the decrease in collagen cross-links and collagen content, which lead to delayed palatal shelf elevation, thus resulting in cleft palate [10,25,26]. However, we thought that this was not the only reason for the delayed palatal shelf elevation caused by *Loxl3* deletion. In the present study, we further clarified that the delayed palatal shelf elevation occurred between E14 and E14.5 in *Loxl3*^−/−^ mice.

Previous studies indicated that there is a correlation between palate development and reduced cell proliferation [12,13]. The palatal shelves elevate to a horizontal position above the tongue between E14 and E14.5 [11,27]. We found that *Loxl3* deletion caused reduced cell proliferation in both medial and posterior palatal mesenchyme, decreased migration of palatal mesenchymal cells, and decreased expression of proliferation-related genes, such as Osr2, Shh, Smo and Fgf10 in vivo and in vitro at E14. However, we examined the cell proliferation of the palate at E14.5, and we found no obvious difference between the *Loxl3*^−/−^ mice and the wild-type mice [10]. This may be because *Loxl3* plays a role in promoting cell proliferation at the initial stage of palatal shelf elevation (E14), but returns to normal after the completion of palatal shelf elevation. Previous studies showed that deletion of Osr2, Fgf10, and Shh all lead to cleft palate during normal palatal development, and they play an important role in the regulation of palatal protrusive cell proliferation [19,20,21,28]. It has been shown that loss of Osr2 causes delay in palatal shelf elevation, resulting in cleft palate [21]. Shh-Smo signaling can positively regulate the expression of Osr2 during palatal protrusive development [3,29,30]. Shh and Fgf10 affect cell migration in mice [31,32]. These genes interact with each other to regulate the mouse palatogenesis process [17,30]. We also observed that *Loxl3*^−/−^ mice had lower palatal mesenchymal cell migration ability than wild-type mice at E14, which may be due to decreased expression of Fgf10 and Shh. This phenomenon may be related to the effect of *Loxl3* loss on palate fusion, affecting palate elongation and fusion by affecting cell migration. Reduced cell migration may affect the soft palate elongation and palatal shelf fusion. Thus, we suggested that loss of *Loxl3* results in decreased expression of proliferation-related genes, which in turn cause decreased cell proliferation and ultimately exacerbates the delayed palatal shelf elevation. Cell migration are also necessary for the development of the palate [14]. No relationship between cell migration and palate elevation has been found, and we hypothesized that reduced cell migration caused by *Loxl3* loss may affect subsequent elongation and fusion.

In our study, we found that *Loxl3* deletion mainly caused reduced cell proliferation in the palatal mesenchyme. In order to further explore the role of *Loxl3* in the palatal mesenchyme, we designed and used Col2a1-Cre to obtain *Loxl3* conditional knockout mice in the palatal mesenchyme. We found that this conditional knockout also caused reduced cell proliferation, reduced collagen deposition and delayed palatal shelf elevation but normal palatal shelf fusion. Studies have shown that collagen exists widely in various stages of palate development, and the normal distribution of collagen fibers is of great significance for the palatal shelf elevation [16,33]. Collagen cross-linking is important in palatal shelf elevation, which significantly affects cell proliferation and migration [33,34]. Collagen maintains the elasticity and integrity of the palate and facilitates the elevation and fusion of the palate [35]. So we considered that *Loxl3* deletion affected the production of immature bivalent cross-linking of collagen fibers [10], thus affecting cell proliferation and migration. We proposed that Loxl3 deficiency causes delayed palatal shelf elevation mainly by affecting the proliferation and collagen deposition of palatal mesenchymal cells during the palatal shelf elevation phase. However, the palatal shelf fusion was completed due to the recovery of cell proliferation (no change in cell proliferation at E14.5 in previous studies [10]) and the accumulation of collagen deposition (difference between partial and complete *Loxl3* knockout). However, it is not clear how a gene involved in crosslinking of collagen regulates cell proliferation of mesenchymal cells. We believe that loss of loxl3 may reduce cell proliferation through mechanisms that are currently unclear. This is one of the directions for our next investigation.

In our study, we noticed that the loss of *Loxl3* affects the expression of Shh, Fgf10 and Smo. Previous studies showed that the SHH-FGF10 signaling pathway plays a role in palatal shelf fusion, and SHH and FGF10 form a critical positive feedback loop in early palate growth [30,36]. Loss of Shh function severely disrupted the growth of the palate and resulted in failure to achieve midline contact of the palate [36]. Reduced Smo expression reduces palatal shelf fusion [37]. Loss of *Fgf10* can affect normal palate development, *Fgf10*^−/−^ mice have anomalous fusion of the palatal shelves [38]. Moreover, many types of collagen (type I, III, V, VI, VII, IX, XII) play an important role in the palatal shelf fusion stage [16]. So we hypothesized that Loxl3 might affect palatal shelf fusion by affecting Shh-Smo signaling expression or other types of collagen assemble. There are several possible effects of Loxl3 on palatal shelf fusion, which still need to be confirmed by further experiments. We have not fully elucidated the mechanism by which Loxl3 causes cleft palate, and not elucidated in detail the reasons for other genetic alterations caused by Loxl3 deletion. We believe that the mechanism of Loxl3 affecting cleft palate can be studied to help Stickler patients and other patients with cleft palate in clinical practice.

In conclusion, we investigated the mechanism of delayed palatal shelf elevation caused by *Loxl3* ablation. *Loxl3* deficiency may affect the cell proliferation and collagen deposition of palatal mesenchyme by affecting the expression of proliferation-related genes, and then leading to delayed palatal shelf elevation.

## 4. Materials and Methods

### 4.1. Mice

Col2a1-Cre mice were obtained from Jackson Laboratory, Bar Harbor, ME, USA. Homozygous *Loxl3*-deficient mice (*Loxl3*^−/−^) and *Loxl3*-floxed mice (*Loxl3^f/f^*) were generated as previously described [10]. *Col2a1-Cre* mice were crossed with *Loxl3^f/f^* mice to generated *Col2a1-Cre; Loxl3^f/f^* mice which were chondrocyte-specific *Loxl3* knockout mice. *Rosa26-tdTomato* males were crossed with *Col2a1-Cre* females to obtained *Col2a1-Tomato* mouse embryos of different embryonic stages. 

### 4.2. Histological Analysis

Embryos were isolated at different stages, fixed overnight in 10% neutral buffered formalin and embedded in paraffin. Coronal sections of hearts and transverse sections of lungs, aortae and tracheae were stained with haematoxylin and eosin (H&E). Coronal sections of the anterior region of palate and eyes and sagittal sections of spinal columns were stained with H&E, Sirius red or Masson’s trichrome, Photomicrographs of Sirius red (Ruitaibio, Beijing, China) or Masson’s trichrome (Yuanyebio, Beijing, China) were analyzed with NIH image analysis software (ImageJ Version 1.48 V, National Institutes of Health, Bethesda, MD, USA). The area of collagen fibers was calculated as the ratio of the integral optical density of collagen fibers to the total area [10].

### 4.3. BrdU Labeling and Proliferation Analysis

Pregnant mice were intraperitoneally injected with BrdU (cat. no. B5002, Sigma-Aldrich, Saint Louis, MO, USA) at 100 μg/g of body weight for two hours before sacrificed. The embryos were isolated, fixed with 4% paraformaldehyde and dehydrated with 15% and 30% sucrose solutions. The tissue were embedded in OCT and sectioned at 8 μm. BrdU detection was performed according to the manufacturer’s manual (Sigma-Aldrich).

### 4.4. RNA Extraction and Quantitative Real-Time RT-PCR

The total RNA of fetal palate was extracted using Trizol method and stored at −80 °C. The reverse transcription reaction system follows the manual of the reverse transcription kit (Takara Bio Inc., Shiga, Japan). The following primers were used: Osr2 forward (5′-TCTTTACACATCCCGCTTCC-3′) and reverse (5′-GGAAAGGTCATGAGGTCCAA-3′); Pax9 forward (5′-TATTCTGCGCAACAAGATCG-3′) and reverse (5′-GGTGGTGTAGGCACCTTAGC-3′); Shh forward (5′-AGGAACTCACCCCCAATTACAAC-3′) and reverse (5′-AGAGATGGCCAAGGCATTTAACT-3′); Smo forward (5′-GAGCGTAGCTTCCGGGACTA-3′) and reverse (5′-CTGGGCCGATTCTTGATCTC-3′); Fgf10 forward (5′-CAGTAGAAATCGGAGTTGTTGCC-3′) and reverse (5′-TGAGCCATAGAGTTTCCCCTTC-3′).

### 4.5. Western Blot Analysis

Palatal shelf tissues of mice embryos were lysed on ice in lysis buffer containing RIPA (Thermo, Waltham, MA, USA) and PMSF (the ratio of RIPA to PMSF is 100:1). The tissue homogenate obtained by the homogenizer was centrifuged for 13 min at 12,000 rpm and 4 °C. The supernatant was taken for Western blot analysis. Western blotting was performed according to standard protocols. The primary antibodies that were used were mouse anti-Osr2 (1:1000, Santa Cruz, Dallas, TX, USA), rabbit anti-Shh (1:1000, BOSTER, Wuhan, China), anti-Smo (1:1000, Abcam, Waltham, MA, USA), anti-Fgf10 (1:400, BOSTER, China), rabbit anti-Pax9 (1:1000, Elabscience, Wuhan, China), mouse anti-Brdu antibodies (1:400, Proteintech, Rosemont, IL, USA) and rabbit anti-β-actin antibodies (1:5000, Abmart, Shanghai, China). The secondary antibodies that were used were goat anti-rabbit IgG-HRP (1:5000, ZSGB-BIO, Beijng, China) and goat anti-mouse IgG HRP (1:5000, ZSGB-BIO, China). The protein bands were quantified using NIH image analysis software (ImageJ Version 1.48V, National Institutes of Health, USA).

### 4.6. Cell Culture

Palatal shelves of E14 mouse embryos were removed and digested at 4 °C for 8–12 h with Dispase II diluted to in complete medium. Epithelial cells and mesenchymal cells of the palatal shelf were isolated. The mesenchymal cells were digested with 0.25% trypsin. Digestion was terminated with complete medium. The supernatant was discarded after centrifugation at 1200 rpm for 5 min. The cells were re-suspended in complete medium and cultured at 37 °C and 5% CO_2_.

### 4.7. Immunofluorescence

The cells and tissues of the palate were stained with immunofluorescence using standard staining procedures [10] with the following primary antibodies: mouse anti-PCK (1:200, BOSTER, China), mouse anti-Vimentin (1:200, Cell Signaling, Danvers, MA, USA), rabbit anti-type II collagen (1:200, Abcam, USA), rabbit anti-LOXL3 (1:400, American Research Products, Belmont, MA, USA), mouse anti-Fibronection (1:200, BD, Franklin Lakes, NJ, USA). The secondary antibodies that were used were rhodamine (TRITC)-conjugated AffiniPure goat Anti-mouse IgG (H+L) (1:200, ZSGB-BIO, China), fluorescein-conjugated AffiniPure goat anti-rabbit IgG (H+L) (1:200, ZSGB-BIO, China), fluorescein-conjugated AffiniPure goat anti-mouse IgG (H+L) (1:200, ZSGB-BIO, China), rhodamine (TRITC)-conjugated AffiniPure goat anti-rabbit IgG (H+L) (1:200, ZSGB-BIO, China).

### 4.8. Statistical Analysis

Data are expressed as the mean ± SEM. Differences in the measured variables between experimental and control groups were assessed using Student’s *t*-test. Differences were considered statistically significant at *p* < 0.05.

## Data Availability

The original contributions presented in the study are included in the article/Supplementary Materials. Further inquiries can be directed to the corresponding authors.

## Supplementary Materials

The supporting information can be downloaded at: https://www.mdpi.com/article/10.3390/ijms26104815/s1.
