# Supplementary material for: Loxl3 Affects Palatal Shelf Elevation by Regulating Cell Proliferation and Collagen Deposition
Source: Int J Mol Sci. 2025 May 17;26(10):4815. doi: 10.3390/ijms26104815 (PMC12111807; doi:10.3390/ijms26104815)
Supplement: Supplementary file 1 [file ijms-26-04815-s001.zip › ijms-3566556-supplementary.pdf]

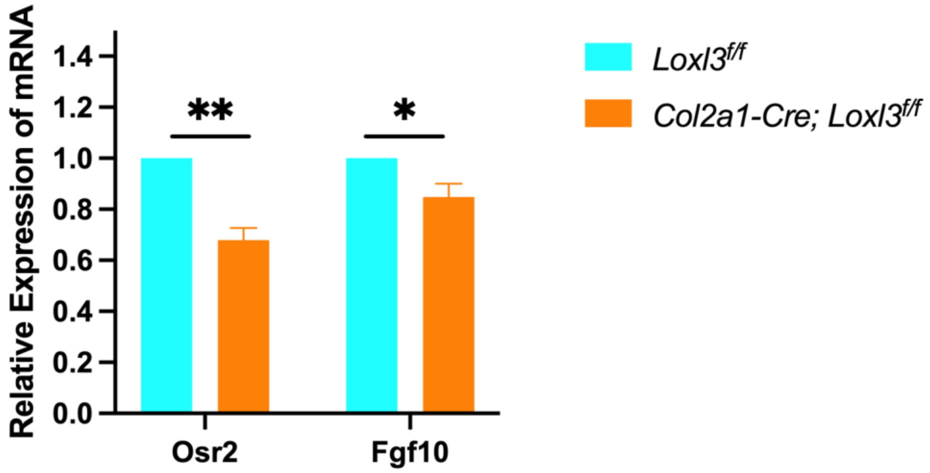

Supplemental Figure S1 Comparison of *Osr2* and *Fgf10* mRNA expression **i**n primary palatal mesenchymal cells of E14. n = 5, **P* < 0.05, ***P* < 0.01, mean ± SEM.

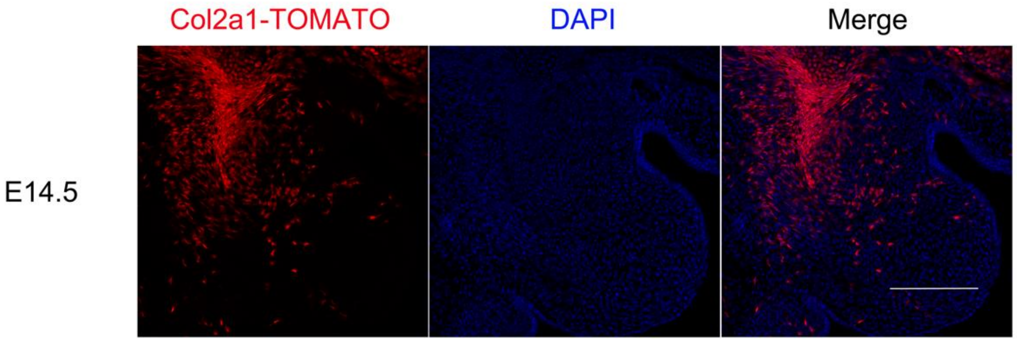

Supplemental Figure S2 The Cre recombinase expression under the Col2a1 promoter in palatal shelves at E14.5. The expression of Cre recombinase (red) was strongly positive in mesenchyme of palatal shelves. Bar: 200 µm.
